# Guillain–Barré syndrome with bilateral facial diplegia secondary to severe acute respiratory syndrome coronavirus-2 infection: a case report

**DOI:** 10.1186/s13256-021-03120-w

**Published:** 2021-11-15

**Authors:** Natalia Ramirez, David Ujueta, Luis Felipe Diaz, Lucila Emilse Folleco, Andrea Rodríguez, Ivan Gaona, Mauricio O. Nava-Mesa

**Affiliations:** 1 Department of Neurology, Fundación Cardioinfantil (FCI), 110131 Bogotá, Colombia; 2 Departamento de Medicina Física y Rehabilitación,, Departamento de Neurofisiología, FCI, 110131 Bogotá, Colombia; 3 Escuela de Medicina y Ciencias de la Salud, GI en Neurociencias-NeURos, Universidad del Rosario, 111221 Bogotá, Colombia

**Keywords:** COVID-19, SARS-CoV-2, Guillain–Barré syndrome, Facial diplegia

## Abstract

**Background:**

The new coronavirus, known as severe acute respiratory syndrome coronavirus 2 (SARS-CoV-2) owing to its similarity to the previous severe acute respiratory syndrome (SARS), is characterized by causing, in most patients, nonspecific symptoms similar to those of the common flu. It has been reported that many coronavirus disease 2019 (COVID-19) patients presented neurological symptoms that involve the central and peripheral nervous systems. In addition, there have been several reports of patients who presented Guillain–Barré syndrome related to  COVID-19 , with sensory and motor compromise in the extremities.

**Case presentation:**

In this report, we describe a rare case of Guillain–Barré syndrome in a 50-year-old Hispanic male with bilateral facial palsy as the only neurological manifestation, following SARS-CoV-2 infection. A complete neurophysiological study showed severe axonal neuropathy of the right and left facial nerves.

**Conclusion:**

Regardless of severity, clinicians must to be aware of any neurological manifestation generated by COVID-19 and start performing more neurophysiological tests to determine if the infection induces an axonal, myelin, or mixed involvement of the peripheral nervous system.

**Supplementary Information:**

The online version contains supplementary material available at 10.1186/s13256-021-03120-w.

## Introduction

The new coronavirus, known as severe acute respiratory syndrome coronavirus 2 (SARS-CoV-2) owing to its similarity to the previous severe acute respiratory syndrome (SARS), is a highly pathogenic and rapidly spreading virus [1]. Around 36% of patients in a Wuhan hospital presented neurological symptoms related to the virus, compromising the central nervous system (CNS), peripheral nervous system (PNS), and skeletal muscle [2].

There have been several reports of patients who presented Guillain–Barré syndrome (GBS) related to COVID-19 with neurological affectation days or weeks after respiratory symptoms [3]. The prevalence is unclear and the total number of patients with concurrent GBS and COVID-19 remains unknown as more cases are published every day. Moreover, despite most of the patients having the typical motor/sensory syndrome, there are also case reports of other variants [4]. To our knowledge, 73 cases of adult patients suffering from GBS and SARS-CoV-2 infection [5–9] have been reported, most of them presenting motor and sensory syndrome.

To date, there are 17 reported cases in the literature of patients with GBS and facial nerve palsy associated with SARS-CoV-2 infection, most of them with limb compromise (that is ascending weakness, areflexia in the lower extremities, unsteady gait) and sensory symptoms (that is, ascending paresthesias, reduced sensation to pinprick) [6, 10, 11]. Only one case reports bilateral facial palsy without other neurological findings [10]. In the present case report, we describe a patient previously diagnosed with COVID-19 who attended our hospital (Fundación CardioInfantil—FCI) with bilateral facial nerve palsy, a variant of Guillain–Barré syndrome known as facial diplegia. Given that previous reports of COVID-19-associated GBS have greater sensory and motor compromise in extremities, the present case has the particularity of having only bilateral facial nerve compromise. In contrast to previous reports of facial diplegia induced by SARS-CoV-2 infection, we included a complete neurophysiological test to characterize the type of nerve injury.

## Case report

### Clinical history

On 1 May 2020, a previously healthy 50-year-old Hispanic male presented with a 2-day history of slurred speech, with particular difficulty in the pronunciation of some consonants and closed vowels, trouble closing eyes, and bilateral facial droop. He reported no numbness, extremity weakness, inability to swallow, or blurry vision, among other neurological manifestations. He had had contact with his father, who died of SARS-CoV-2 infection 1 month prior to the admission. Around the time of this contact, he presented asthenia, ageusia, and hyporexia with two positive reverse-transcription polymerase chain reaction (RT-PCR) results for SARS-CoV-2 infection, the first one 19 days prior to the onset of neurological symptoms and the second one 6 days before arriving at the emergency department. He received management with hydration and paracetamol for symptoms such as asthenia, adynamia, and nasal congestion. He was not taking any medication prior to diagnosis, and he did not smoke or consume alcohol. He denied exposure to environmental toxins or chemicals and was unemployed.

**Fig. 1:**
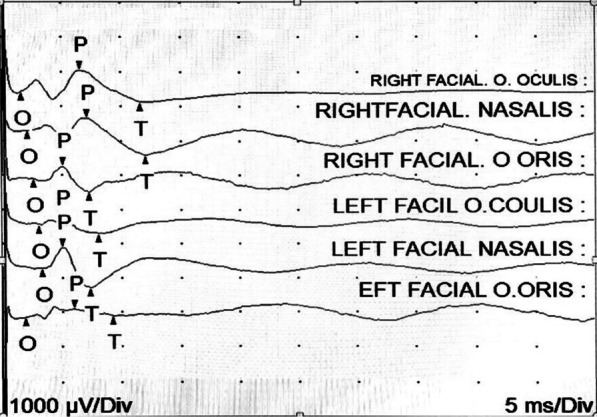
Facial nerve conduction study showing reduction in the compound muscle action potential (CMAP) amplitude bilaterally

On admission, his vital signs were normal (pulse 78 beats per minute, blood pressure 130/80 mmHg, temperature 36 °C), as was the general physical examination. Because the patient had not signs of respiratory distress (saturation ≥ 90%), thoracic X-rays were not taken. Neurological examination revealed mild dysarthria and bilateral facial palsy (House–Brackmann grade III). No other cranial nerve abnormalities were found. There were no meningeal signs. He had normal gait without limb weakness or areflexia. Blood cell count showed lymphopenia (199 cells/µL) without any other relevant findings. Non-contrasted and contrasted brain MRIs were performed without pathological findings. A lumbar puncture was done at day 6, with the presence of albuminocytologic dissociation (0 cells, proteins 210 mg/dL). Cerebrospinal fluid (CSF) Gram stains and culture were negative. No antiganglioside antibodies were available at our institution. The same day, neurophysiological studies (electromyography and nerve conduction) reported severe acute axonal neuropathy of both right and left facial nerves, with prolongation of latency and severe amplitude reduction in all the motor potentials recorded bilaterally. Also, a drastic reduction was recorded in recruitment patterns in facial muscles, with interference patterns less than 30%. All those changes suggest denervation (see Tables 1, 2, Figure 1 for details). Motor and sensory conduction studies in the upper and lower extremities were normal (see Additional file 1 for details-supplementary material).

**Table 1:** Electromyography study showing significant reduction in recruitment patterns in the orbic oris, orbic oculi, and nasalis muscles bilaterally. No fibrillation potentials or fasciculation were found

Side	Muscle	Nerve	Root	Ins Act	Fibs	Psw	Amp	Dur	Poly	Recrt	Int Pat	Comment
Right	Medgastroc	Tibial	S1-2	Nml	Nml	Nml	Nml	Nml	0	Nml	Nml	–
Right	AbdHaluccis	Med. Plantar	S1-2	Nml	Nml	Nml	Nml	Nml	0	Nml	Nml	–
Left	Orbic oris	Facial	CN VII	Nml	Nml	Nml	Nml	Nml	0	Reduced	25%	–
Left	Orbis oculi	Facial	CN VII	Nml	Nml	Nml	Nml	Nml	0	Reduced	25%	–
Left	Nasalis	Facial	CN VII	Nml	Nml	Nml	Nml	Nml	0	Reduced	25%	–
Left	Masetero	Trigémino		Nml	Nml	Nml	Nml	Nml	0	Nml	Nml	–
Right	Orbic oris	Facial	CN VII	Nml	Nml	Nml	Nml	Nml	0	Reduced	25%	–
Right	Orbis oculi	Facial	CN VII	Nml	Nml	Nml	Nml	Nml	0	Reduced	25%	–
Right	Nasalis	Facial	CN VII	Nml	Nml	Nml	Nml	Nml	0	Reduced	25%	–
Right	Masetero	Trigémino		Nml	Nml	Nml	Nml	Nml	0	Nml	Nml	–
Right	Vastus Lat	Femoral	L2-4	Nml	Nml	Nml	Nml	Nml	0	Nml	Nml	–
Right	Ext Dig Bre	Dp Br Peron	L5,S1	Nml	Nml	Nml	Nml	Nml	0	Nml	Nml	–
Left	1stDorInt	Ulnar	C8,T1	Nml	Nml	Nml	Nml	Nml	0	Nml	Nml	–

Motor units had normal morphology with an adequate recruitment pattern in the exploration of proximal and distal muscles in the upper and lower extremities

*Dp Br Peron* deep peroneal nerve;* DorInt* dorsal interosseous;* Ext Dig Bre* extensor digitorum brevis;* Nml* normal

**Table 2:** Nerve conduction studies showing abnormal facial nerve conduction, compatible with severe axonal neuropathy of the right and left facial nerve of acute evolution with involvement of its temporal, zygomatic, buccal, and mandibular branches

Site	NR	Onset (ms)	Norm Onset (ms)	O-P A P (mV)	P-T Am P (mV)	Norm O-P Am p	Site 1	Site 2	Delt a-0 (ms)	Dist (cm)	Vel (m/s)	Norm Vel (m/s)
Right facial O. oculis		4.4	< 4.2	0.9	0.8	> 5	Right facial nasalis	Right facial O. oculis	0.7			> 50
Right facial nasalis		5.1		0.5	1.1		Right facial O. oris	Right facial nasalis	1.3	0.0		
Right facial O. oris		3.8		0.4	0.8		Left facial O. oculis	Right facial O. oris	0.8	0.0		
Left facial O. oculis		3.0		0.0	0.3							
Left facial nasalis		4.0		0.5	1.2							
Left facial O. oris		4.3		0.1	0.1							

Prolonged latency and severe amplitude reduction in all the motor potentials were recorded bilaterally in the O. oculis, nasalis, and O. oris muscles.

### Outcome and follow-up

In accordance with several guidelines for management of GBS [12, 13], plasmapheresis was offered to the patient by our neurology service. However, the patient declined the procedure for personal reasons. The patient was monitored in hospital for 8 days without developing motor symptoms or additional neurological deterioration and without receiving immunomodulatory treatment. He was discharged with a physical rehabilitation program. At a follow-up performed 30 days after discharge, the patient presented complete recovery.

## Discussion

We presented the case of a previously healthy 50-year-old Hispanic male recently diagnosed with SARS-CoV-2 infection, who was admitted to the hospital without neurological manifestations except for symptoms and signs of bilateral facial nerve palsy. According to the history and physical examination, he was diagnosed with bilateral facial diplegia, an uncommon variant of GBS. Albuminocytologic dissociation was found on CSF study. Brain images were assessed with no pathological findings. Interestingly, the GBS-related symptoms he presented were only facial, but in contrast with previous reports, we included a neurophysiology study that reported severe acute axonal neuropathy of the right and left facial nerves with prolongation of latency and severe reduction of the amplitude in all the motor potentials recorded bilaterally.

Guillain–Barré syndrome (GBS) is an inflammatory disease of the peripheral nervous system. Its manifestations typically involve weakness and sensory symptoms that begin in the lower extremities and progressively ascend until the arms and face are compromised [14]. It has been proposed that a previous infection generates an immune response, which in turns cross-reacts with peripheral nerve components because of its molecular mimicry, resulting in acute polyneuropathy with axonal and/or myelin involvement [14, 15]. Diagnosis is made taking into account the patient’s clinical history and neurological examination, and is associated with changes in the CSF such as albuminocytologic dissociation (CSF protein > 0.55 g/L with no increase in white blood cell count) or electrophysiology studies, including F-wave and H-reflex suggesting peripheral nerve compromise. These features are important for the differentiation of different subtypes of GBS [14].

In addition to the typical clinical presentation described previously, other less frequent GBS variants have been described, including bilateral facial nerve palsy [16], as in the case of the above-mentioned patient. Up to 10% of GBS patients present facial weakness as the main clinical manifestation of this disease [17], and 20–60% of the cases with motor symptoms have reported bilateral facial involvement [18]. Other manifestations documented in patients with GBS and bilateral facial palsy were sensory symptoms including paresthesias and reduced perception of pinprick, and motor involvement with areflexia and weakness [6, 7]. Less common symptoms were positive Romberg sign and hypogeusia [6].

As mentioned previously, around 73 cases of GBS related to SARS-CoV-2 infection have been reported [5–9], mostly with respiratory symptoms prior to neurological impairment. Other variants included Miller Fisher, acute motor axonal neuropathy (AMAN), and bilateral facial nerve palsy, as in the case of our diagnosed patient. Seventeen cases of facial involvement variants have been reported in the literature. Of these, one presented bifacial weakness as the only neurological manifestation [10] while others were described as motor or sensory symptoms followed by facial diplegia [6]. In one case, facial palsy preceded motor symptoms [19], and in the others, the facial palsy and weakness or paresthesias developed at the same time [6, 11]. Recently, a case of Bell’s palsy on the left side of a 65-year-old woman in China associated with SARS-CoV-2 infection was reported, though without preceding fever, cough, or any other respiratory symptoms. The patient recovered after antiviral treatment; however, no neurophysiological or CSF test was performed [20].

It is already known that GBS could be triggered by prior bacteria or virus infections, such as *Campylobacter jejuni*, influenza virus, cytomegalovirus, or Epstein–Barr virus [14]. Taking into account the revised literature and the temporal relationship between the infection and the onset of symptoms described in our case, it is possible that the pathophysiology of SARS-CoV-2 causing GBS via inflammatory reaction through immune mimicry is similar to that proposed for other pathogens [21, 22]. However, this should be further characterized with the continuing and growing evidence on the implications of COVID-19 in this pathology.

## Conclusion

SARS-CoV-2 should be considered as a potential trigger of GBS and all its variants. Clinicians should be aware of this possible risk when treating a COVID-19-positive patient, independently of the severity of their respiratory condition.

Considering the large number of neurological disorders induced directly or indirectly by SARS-CoV-2, a neurological physical examination should be performed routinely in these types of patients, especially in those with severe symptoms. It is important to start performing more clinical neurophysiology tests to record these neurological alterations and determine if the virus generates an axonal, myelin, or mixed involvement of the PNS.

## Supplementary Information

**Additional file 1.** Supplementary material.

## Data Availability

The datasets used and/or analyzed during the current study are available from the corresponding author on reasonable request.

## Abbreviations

AMAN: Acute motor axonal neuropathy
CNS: Central nervous system
CSF: Cerebrospinal fluid
GBS: Guillain–Barré syndrome
PNS: Peripheral nervous system
MRI: Magnetic resonance imaging
SARS: Severe acute respiratory syndrome
