# Supplementary material for: Guillain–Barré syndrome with bilateral facial diplegia secondary to severe acute respiratory syndrome coronavirus-2 infection: a case report
Source: J Med Case Rep. 2021 Nov 15;15:558. doi: 10.1186/s13256-021-03120-w (PMC8591425; doi:10.1186/s13256-021-03120-w)
Supplement: Supplementary file 1 — Additional file 1. Supplementary material. [file 13256_2021_3120_MOESM1_ESM.docx]

**Supplementary material**


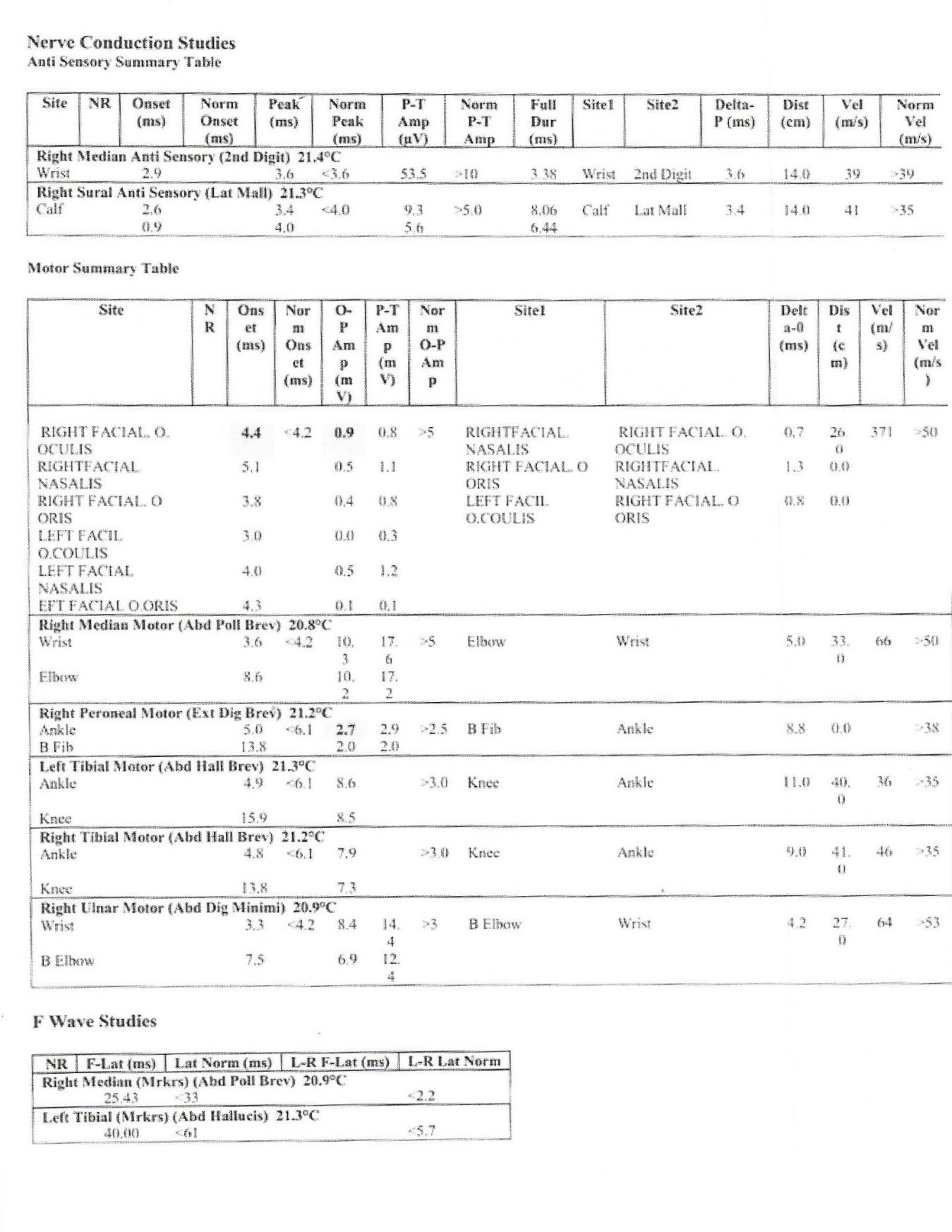


**Neurophysiological study**

Findings:

Motor and sensory nerve conduction studies of the right upper limb:

1. Motor action potentials of median and ulnar nerves with normal conduction amplitudes and latencies

2. Sensory action potential of the median nerve with normal latency and amplitude.

3. F waves of the right median nerve, with persistence in 100% of the recording and normal latencies.

Motor and sensory nerve conductions studies of the lower limbs:

1. Motor action potential of the right fibular nerve with normal latencies and amplitudes.

2. Motor action potentials of tibial nerves with normal latencies and amplitudes

3. Sensory action potential of the right superficial fibular nerve with normal latency and amplitude.

4. F wave of the right tibial nerve with persistence in 100% of the records, normal latencies
